# Scoliosis detection, patient characteristics, referral patterns and treatment in the absence of a screening program in Norway

**DOI:** 10.1186/1748-7161-7-18

**Published:** 2012-10-25

**Authors:** Raphael Dziwornu Adobor, Rolf Bjarne Riise, Roger Sørensen, Thomas Johan Kibsgård, Harald Steen, Jens Ivar Brox

**Affiliations:** 1 Section for Spine Surgery, Department of Orthopedic Surgery, Oslo University Hospital- Rikshospitalet, Sognsvannsveien 20, Oslo, 0372, Norway; 2 Biomechanics lab, Department of Orthopaedic Surgery, Oslo University Hospital-Rikshospitalet, Sognsvannsveien 20, Oslo, 0372, Norway

## Abstract

**Background:**

Early diagnosis of idiopathic scoliosis allows for observation and timely initiation of brace treatment in order to halt progression. School scoliosis screening programs were abolished in Norway in 1994 for lack of evidence that the programs improved outcome and for the costs involved. The consequences of this decision are discussed.

**Objectives:**

To describe the detection, patient characteristics, referral patterns and treatment of idiopathic scoliosis at a scoliosis clinic during the period 2003–2011, when there was no screening and to compare treatment modalities to the period 1976–1988 when screening was performed.

**Methods:**

Patient demographics, age at detection, family history, clinical and radiological charts of consecutive patients referred for scoliosis evaluation during the period 2003–2011, were prospectively registered. Patients were recruited from a catchment area of about 500000 teenagers. Maturity was estimated according to Risser sign and menarcheal status. Severity of pain was recorded by a verbal 5-point scale from no pain to pain at all times. Physical and neurological examinations were conducted. The detector and patient characteristics were recorded. Referral patterns of orthopedic surgeons at local hospitals and other health care providers were recorded. Patient data was obtained by spine surgeons. Treatment modalities in the current period were compared to the period 1976–1988.

**Results:**

We registered 752 patients with late onset juvenile and adolescent idiopathic scoliosis from 2003–2011. There were 644 (86%) girls and 108 (14%) boys. Mean age at detection was 14.6 (7–19) years. Sixty percent had Risser sign ≥ 3, whilst 74% were post menarche with a mean age at menarche of 13.2 years. Thirty-one percent had a family history of scoliosis. The mean major curve at first consultation at our clinic was 38° (10°-95°). About 40% had a major curve >40°. Seventy-one percent were detected by patients, close relatives, and friends. Orthopaedic surgeons referred 61% of the patients. The mean duration from detection to the first consultation was 20(0–27) months. The proportion of the average number of patients braced each year was 68% during the period with screening compared to 38% in the period without screening, while the proportion for those operated was 32% and 62%, respectively ( p=0.002, OR 3.5, (95%CI 1.6 to 7.5).

**Conclusion:**

In the absence of scoliosis screening, lay persons most often detect scoliosis. Many patients presented with a mean Cobb angle approaching the upper limit for brace treatment indications. The frequency of brace treatment has been reduced and surgery is increased during the recent period without screening compared with the period in the past when screening was still conducted.

## Background

Idiopathic scoliosis is a complex three dimensional deformity of the spine characterized by a lateral deviation and axial rotation [1-3]. Classification is according to the age of onset; infantile, from birth to 3 years; juvenile from 3 to 8 years, and adolescent from 10 years to maturity [4]. Idiopathic scoliosis is also classified into early onset (<5 years) or late onset (>5years) [5]. Adolescent idiopathic scoliosis (AIS) is the most common form and is often associated with rapid growth [4].The prevalence rate of idiopathic scoliosis as proposed by the Scoliosis Research Society (Cobb angle >10°) [6] is reported from 0.5% to 3%, but only 5% of these patients have curve progression to >30° [7,8]. We recently performed screening in 4000 twelve years old Norwegian children and found a 0.55% point prevalence of AIS [9]. The ratio of girls to boys is equal for minor curves, but rises for girls as the curve magnitudes, reaching a ratio of 1:8 for those requiring treatment [10].The etiology is not known, but several genetic predisposing factors have been described [11-16].

Early detection of scoliosis allows for monitoring of the development and progression of the deformity, and timely initiation of bracing that is reported to be effective by non-randomised studies [17-21], although the scientific evidence is weak according to a recent Cochrane Review [7]. After scoliosis has been detected, skeletal growth (Risser sign 0 and 1) gender and curve location has consistently been reported to increase the risk of progression [2,4,22-24], while conflicting risk is reported for curve magnitude with some studies reporting larger curves increasing the risk of progression, whilst some studies do not [10]. Curve flexibility (initial correction in brace) is reported to reduce the risk of progression in braced patients [25]. Progression is most rapid during peak skeletal growth, which precedes menarche in girls and occurs 6 to 12 months after the onset of axillary and facial hair in boys [26]. Several methods have been applied to estimate skeletal age including the Risser sign and Greulich and Pyle radiographic atlas [27]. The Risser sign is the most common method used to assess remaining growth in patients with idiopathic scoliosis [28].

Bracing to prevent or limit scoliosis progression is usually recommended for progressive curves >25°[19]. Surgical treatment is considered for curves >45°-50° to limit further progression, and correct the deformity [29-31]. Screening for scoliosis allows for early detection and has in addition, provided valuable knowledge about prevalence, aetiology and the natural history [32,33]. However, there are objections to scoliosis screening based largely on the low specificity of the screening test and the costs involved because of over-referrals [8,34,35]. In our recently published screening program, we found a specificity of 0.99 and a positive predictive value of 0.37 [9].

Scoliosis was usually detected through school screening program in Norway until 1994. Upon the advice by the United States Preventive Services Task Force, many western countries including Norway, abolished scoliosis screening program in the nineties [36,37]. In the Scandinavian countries, Sweden is the only country with an ongoing scoliosis screening program [19]. In recent years, The Scoliosis Research Society and the American Academy of Orthopaedic surgeons, the Paediatric Orthopaedic Society of North America, and the American Academy of Paediatrics have endorsed scoliosis screening, while The Canadian Task Force on the Periodic Health examination, the British Orthopaedic Association, and the British Scoliosis Society do not recommend screening [38,39].

The effectiveness of scoliosis screening is debated [40-44]. Previous studies have reported that scoliosis patients detected through screening had smaller curves at diagnosis and had lower risk of having surgery [40,41,43,45]. Researchers from the Netherlands reported that screening reduced the need for surgery in a retrospective study [45], but later concluded that screening did not reduce the need for surgery in a case control study [46]. Studies on the impact of discontinuation of school screening program have reported that the majority of cases are detected by family members and friends, and detection was late with regards to benefit from brace treatment [47,48].

The primary objective of the present study was to evaluate the detection of scoliosis and to describe patient characteristics and referral patterns to a scoliosis clinic with regard to brace treatment in the absence of a screening program during the period 2003–2011. The secondary objective was to evaluate the impact of discontinuation of school screening scoliosis on the rate of brace and surgical treatment of scoliosis by comparing the yearly average numbers of brace and surgical treatment in the period 2003–2011 when there was no screening, to the period 1976–1988 when scoliosis screening was still being conducted in Norway.

## Methods

### Study design

The study is a prospective study to describe the characteristics of patients with late juvenile and adolescent scoliosis referred for scoliosis evaluation during the period 2003–2011 and to compare these data with historical control data with respect to the number of patients who were braced or operated during the period 1976–1988 when scoliosis screening was still conducted in Norway.

During the period 2003 to 2011, data was collected by 6 experienced orthopaedic spine surgeons, 4 fellows in spine surgery, and 10 resident orthopaedic surgeons. The patients were recruited prospectively beginning in 2003, and included all new referred patients to the specialist clinic at the orthopaedic department, Oslo University Hospital, Rikshospitalet. Norway has a public universal healthcare system in which waiting times for specialists’ evaluation of paediatric cases are generally at an acceptable level. Rikshospitalet is a tertiary referral centre designated to offer specialized services to the Norwegian population of 4.7million inhabitants. It is estimated that the specialist clinic offered scoliosis services to approximately 80% of the Norwegian population representing about 500.000 teenagers during the study periods [49]. The surgeons filled in a standardised 2 page chart based on patient data from interview, clinical examination and radiological measures. The inter-observer agreement of the methods used for collecting data in the standardized chart has not been evaluated. The inclusion criteria was late-juveniles (7 years and older) and adolescents referred to idiopathic scoliosis evaluation for the first time. Patients with infantile and early-onset juvenile idiopathic, neuromuscular, congenital or syndromic scoliosis were excluded.

Patient records and surgical protocols during the years 1976–1988 were reviewed to estimate the number of late-onset juvenile and adolescent idiopathic scoliosis patients that were treated with Boston brace or operated with Harrington’s rods at the Sophies Minde Hospital. The Sophies Minde Hospital was the name of the orthopedic department of the Rikshospitalet during that period, and provided scoliosis services to the same segment of Norwegian population as at now. The results of brace treatment in these patients were previously reported in 3 publications [50-52]. Indication for bracing was a major scoliotic curve >20° with an observed progression >5° and the Risser sign <3, and the indication for surgery was progressive curves >45°-50° in adolescents with remaining growth [51,52].

#### Demographics

Age at scoliosis detection, at evaluation and at menarche was recorded. Family history of idiopathic scoliosis was obtained from the parents of the affected child based on questioning them if any family member had consulted or been braced or operated for scoliosis. The originator of detection was classified into six groups: 1) the patient himself/herself; 2) parents, family, siblings, grandparents, aunt/ uncles and friends); 3) primary physicians; 4) hospital specialists (orthopedic specialist, radiologist); 5) allied health care provider (physical therapist community health nurse, chiropractor, osteopath and 6) non-health care provider (dressmaker, sports instructor or hair dresser).

#### Referral patterns

Referral patterns of primary physicians, physical therapists community health nurse, chiropractors and hospital specialists to the scoliosis clinic were noted. The duration from time of detection to referral and evaluation was also recorded.

#### Clinical assessment

Physical examination was conducted by observing the patient standing for assessment of asymmetries of the shoulder, ribs, scapula, waist and hips. Shoulder symmetry was assessed by the relative position of both shoulders and recorded as normal, asymmetry < 2 cm or asymmetry > 2 cm. Decompensation of the trunk over pelvis was recorded as positive truncal shift. Coronal balance was measured by the plumb line in cm as the lateralizing position of the cervico-thoracic junction in relation to the left or right of the gluteal cleft. Height and body weight were measured by a nurse for calculation of body mass index (BMI kg/m^2^). In the Adams forward bending position, the thoracic rib cage prominence (angel of thoracic rotation (ATR) or prominence of the paraspinal muscles in the thoracolumbar/lumbar area was measured either in degrees using a scoliometer or in centimeters using a ruler. Half of the patients were measured using the scoliometer and half measured in centimeters. Back pain and perception of fatigue of the back muscles were recorded by a verbal 5-point scale: never had pain, seldom pain, sometimes pain, often pain, always pain.

Neurological assessment was performed by evaluating the spinal reflexes, extremity weakness, hyper-reflexia and abdominal reflexes to eliminate neurological etiology.

#### Radiological measures

Skeletal age was estimated by a radiologist using Risser sign obtained from a postero-anterior radiograph at the level of the iliac crest ossification [28]. The convexity and magnitude of the curves were measured according to the Cobb method from the radiographs of full postero-anterior films by 2 experienced radiologists [53]. Scoliosis was classified by the orthopedic surgeons according to the King Moe types [54]. Interrater agreement of Cobb measurements and King-Moe classifications was not evaluated in the present study.

#### Recommended treatment

Prescribed or recommended treatment up to 6 months follow-up after the first consultation was recorded as further observation, brace treatment, surgery or discharge. We assumed that additional patients had surgery either after brace treatment was initiated or after further follow-up. We therefore also reviewed surgical protocols in order to estimate the actual number of patients who were operated yearly during the period of the study.

### Statistical analysis

Descriptive statistics of mean, median, standard deviation, range, and frequencies were calculated using the Statistical Package for Social Science (SPSS), version 14.0 (SPSS Inc., Chicago, IL. We categorized curve size (10.0° – 24.9°; 25.0° - 34.9°; 35.0° – 39.9°; 40.0° – 44.9°; > 45.0°), BMI (<30 and >30) kg/m^2^, age (7–12 years; 13 and 14 years; 15 and 16 years; 17 years and older), and back pain (absent, seldom, sometimes, often and all the time).We applied multivariable logistic regression to estimate the association between back pain as the outcome variable, with gender, curve size, and BMI as predictor variables. Regression analysis was performed in order to examine the confounding effect of curve size, and BMI on the assumed association between back pain and gender. The Hosmer-Lemeshow test was used to assess the logistic model adequacy. Effect sizes were measured by odds ratio (OR) and 95% confidential intervals were calculated.

One-way ANOVA with correction for multiple tests assuming non-equal variance was performed to analyse differences between originator of detection in relation to age and curve size. In addition, the non-parametric Kruskal –Wallis test was used to test differences in patient treatments, and Risser signs between various originators of scoliosis detection. If significance was observed, the non-parametric Mann–Whitney U test was used to detect differences between two groups of originators.

The average number of patients treated with brace and those operated in the two periods were calculated from the prospective data and the administrative data (surgical protocols). Chi-squared statistics was used to compare whether the number of those treated with brace and those operated in the two periods differ from one another. The proportions of the treatment modalities in the two periods, mean difference between the proportions, odds ratio (OR) and 95% confidential intervals were calculated.

The study was approved by the Ethic Committee of the Oslo University Hospital (reference number 12/11063).

## Results

### Demographics

There were 752 patients, 644 (85%) girls and 108 (14%) boys. The age distribution is shown in Table 1. Mean age at scoliosis detection was 14.6± 2.1 (7–20) years. Mean age for girls was 14.5±2.1 and 15.5± 2.1 for boys. Thirty-one percent had a family history of scoliosis with 9% being first degree (parents or siblings) or second degree relations, respectively.

**Table 1:** Main patient characteristic

**Characteristics**	**Frequency (n)**	**%**
**Gender (n=752)**		
Girls	644	86
Boys	108	14
**Age/years**		
7-12	105	14
13-14	228	30
15-16	292	39
17 and older	127	17
**Scoliosis detector**		
Patient him/herself	50	7
Parents/family/ friends	485	64
Primary physicians	93	12
Hospital specialists	12	2
Allied health care provider	96	13
Non-health care provider	16	2
**Risser sign**^**1**^		
**0**	182	24
1	38	5
2	76	10
3	67	9
4	251	34
5	135	18
**Cobb angle/°**		
10 - 24.9	119	16
25 - 34.9	229	31
35 - 39.9	114	15
40 - 44.9	91	12
>45	199	27
**King Moe Classification**^**2**^		
1	220	32
2	259	37
3	164	24
4	40	6
5	9	1
**Back pain**^**3**^		
Absent	307	42
Seldom	139	19
Sometimes	163	22
Often	93	13
All the time	30	4
**Treatment recommendations**		
Brace	161	21
Surgery	195	26
Further observation	192	26
Discharged	204	27

^1)^Not reported, n= 3 ^2)^Not reported, n= 60 ^3)^Not reported n= 20.

### Originator of scoliosis detection

The originator of detection is shown in Table 1. Seventy-one percent were detected by patients, family members or friends, 27% by health care providers, and 2% by non-health care providers. The distribution of patient maturity, curve sizes and treatment modalities according to the various originators of detection is shown in Table 2.

**Table 2:** Age in years, Risser sign, curve size in degrees, and percentage of treatment modalities according to originator of detection

	**Age**	**Risser sign Median(range)**	**Curve size Mean±SD**	**Treatment modalities**			
	**Mean±SD**			Brace Surgery Observation* Discharged			
Patient him/herself	15.4±1.9	4 (0 to 5)	40.6±15.6	8.0	24.5	24.0	36.0
Parents/family/ friends	14.5±2.0	3 (0 to 5)	38.8±14.5	21.9	27.5	26.7	24.0
Primary physicians	14.8±2.1	4 (0 to 5)	35.8±14.4	18.1	24.5	23.4	34.0
Hospital specialists	15.6±2.4	4 (0 to 5)	39.2±15.8	16.7	25.0	16.7	41.7
Allied health care provider	14.5±2.5	4 (0 to 5)	35.3±13.5	28.1	17.7	24.0	30.2
Non-allied health care provider	14.5±2.1	4 (0 to 5)	38.3±15.8	6.3	31.3	43.8	18.8

* After 6 months observation.

Patients whose scoliosis were detected by parents, family and friends were statistically younger compared to those detected by the patients themselves (p=0.01). Risser sign was lower in patients whose scoliosis were detected by parents, family and friends compared to those detected by patients themselves (p=0.005) or those detected by primary physicians (p=0.019).There were no differences in the curve size according to the originator of detection. Community health nurses and physical therapist detected patients with scoliosis that were suitable for brace treatment more than when the patients detect scoliosis themselves (p=0.005).

### Referral patterns

Sixty-one percent of patients were referred by orthopaedic surgeons, 33% by primary physicians, and 5% by physical therapists community health nurses or chiropractors. The mean duration from detection to referral was 16±16.9 (0–27) months and the mean duration from referral to first consultation at our clinic was 4.0 ± 2.6 months.

### Maturity

The distribution of Risser sign is shown in Table 1. Sixty percent were ≥ Risser 3. Seventy-eight percent of the girls had had menarche at the time of the first visit with 36% of them ≥ 2 years earlier. Mean age at menarche was 13.2±1.2 years.

### Physical examination

Mean body mass index (BMI) was 19.7±3.2 (range 11.9 - 41.0) kg/m^2^. BMI was 19.7±3.2 in girls and 19.9±3.3 in boys. Asymmetries of the shoulders were registered in 52%. Twenty –eight percent had coronal imbalance, but only 10% were > 2 cm. Truncal shift was registered in 35%. The mean ATR was 8.4° in the thoracic region and 7.9° in the lumbar region of those measured with the scoliometer, and 1.5 cm in the thoracic region, and 1.2 cm in lumbar region of those measured in centimeters. All patients had normal neurological examination except one girl who had an abnormal superficial abdominal reflex. A supplementary MRI revealed a Chiari malformation and syringomyelia.

### Back pain

Boys reported more often (75%) that they seldom or never had back pain compared with girls (58%), 7% of the boys reported that they have pain often or almost all the time compared with 18% of the girls. There is a significant association between back pain and girls compared with boys (p = 0.006, OR 2.88, 95%CI (1.36 to 6.19).

### Radiological measures

The mean major curve at first consultation was 37.8°± 14.5° (range 10.95°); 37.8°±14.1°in girls and 37.5°±16.8° in boys. Seventy-five percent of the primary curves had convexity towards the right. The curve magnitude and classification according to King-Moe are shown in Table 1.

### Treatment modalities

At the initial consultation, brace treatment was recommended in 18%, surgery in 20%, 8% were discharged, and 54% were scheduled for further observation. Curve size, age, and Risser sign of these patients are shown in Table 3. After 6 months observation, an additional 3% were recommended for brace treatment, 6% for surgery, 35% were discharged, and 48% recommended further follow-up. The recommended treatment modalities at the first consultation and at 6 months observation are shown in Table 1. This means that at the outpatient clinic the yearly average number of patients recommended for brace was 19, and 22 for surgery. The additional review of surgical protocols reflecting a longer follow-up period revealed that on average, 32 patients were operated yearly during the study period.

**Table 3:** Age in years and Risser sign and curve size in degrees, at first consultation

	**Age**	**Risser sign**	**Curve size**
	**Mean± SD**	**Median(range)**	**Mean±SD)**
Brace treatment	12.8±1.9	0(0 to 4)	36.0±8.7
Surgery	14.4±1.7	3(0 to 5)	58.3±10.9
Further observation	15.0±2.0	4(0 to 5)	32.4±9.8
Discharge	16.2±2.0	5(0 to 5)	25.4±8.1

### Brace and surgical treatment 1976–1988

During the period 1976–1988, an average of 41 patients were treated with the Boston Brace each year [51,52]. According to the surgical protocols, an average of 20 patients was operated each year with Harrington’s rods for late- juvenile or adolescent idiopathic scoliosis during the same period.

### Comparison of treatment modalities

The number of patients treated with brace relative to those operated during 1976–1988 was 41/19 and 20/32 during 2003–2011.The proportion of patients treated with brace during 1976–1988 (68%) was higher than during 2003–2011 (38%) and vice-versa for surgical treatment (p=0.002, OR 3.5 95% (CI 1.6 to 7.5). The mean difference was 30% 95% CI (10 to 47).

## Discussion

The majority of patients detected with scoliosis in the absence of screening were skeletally mature and had curves that were not suitable for brace treatment. According to internationally accepted guidelines brace treatment is recommended in growing children with progressive curves >25°, who has at least one year growth potential [19]. The mean chronological age at detection in the present study was 14 years, which is 2 years older than the ideal age [19,55]. At the first presentation, 60% had Risser sign ≥3, and 78% of the girls were post menarche, indicating that most patients were detected late, and not suitable for brace treatment. A large proportion of patients also had curves ≥40° at first presentation which is beyond the international recommendation for initiation of brace treatment [43]. The present results are in agreement with two previous studies that have assessed the impact of discontinuation of scoliosis program on detection, referral patterns and management. In a Canadian study, 32% of patients with AIS were referred too late with regard to brace treatment [48] and in a study conducted in London, United Kingdom, 56% of cases were detected when the primary curve was >40° and not suitable for brace treatment [47].

About 71% of cases were detected by non health care providers either by patients themselves, close family members or friends. It has been suggested that parents should be educated to perceive asymmetries of the back, shoulders, waistline, hips, and breast in their children as early signs of scoliosis and seek early and appropriate medical evaluation [42,48]. In the present study, only 27% of cases were detected by healthcare providers. We found statistical differences in patient maturity, and treatment modalities between different originators of scoliosis detection. Close family members and friends detected patients with scoliosis at a younger age compared to scoliosis detected by the patients themselves. Since majority of curves were large at detection, we found no differences in the curve size according to the originator of detection.

Community health nurses and physical therapists detected patients with scoliosis that were suitable for brace treatment more than when the patients detect scoliosis themselves.

### Referral patterns

Two thirds of all patients were first observed by orthopedic surgeons at local hospitals on average 16 months before being referred and only 1/3 of patients were referred directly by primary physicians or physical therapists or community health nurses to the specialist clinic. Norway, with its public universal healthcare system, promotes referrals to specialized care and waiting times are generally at an acceptable level for a specialist’s evaluation of pediatric cases as shown in this study where the mean waiting time from referral to specialist evaluation was 4 months. A general guideline for primary physicians is to refer suspected cases of AIS to local orthopedic surgeons for diagnosis or refer directly to specialist evaluation of high risk patients for progression. Orthopedic surgeons at local clinics are authorized to diagnose and observe AIS patients for progression and to refer without unnecessary delay progressive curves >20° in immature patients to specialist clinics for evaluation. These orthopedic surgeons downstream the specialists’ clinics are however not authorized to prescribe brace treatment. There is either a late detection of AIS in the community in the absence of screening, or there is a delay in the referral practices of health care providers to specialist evaluation due to their non- awareness of existing guidelines, or failure of education on these guidelines. The result is that many patients with AIS in the present study were referred when mature, and with curves approaching the upper limit of brace treatment indications.

To ensure uniformity and quality of care for scoliosis patients, we suggest that persons involved in child health care like physical therapists community health nurses, chiropractors, sports instructors, primary physicians and orthopedic surgeons be better informed about guidelines for scoliosis detection. This includes the examination of asymmetries of the back in the routine examination of the child [48]. Without objective screening test, proper training and clear guidelines for referrals, there is a risk of inappropriate referrals to the specialized care setting if one will suggest that community health nurses, physical therapists, chiropractors, sports instructors, refer patients directly to scoliosis specialist evaluation without first referring to local orthopedic surgeons. However, the magnitude of the eventual inappropriate referrals is not known. Local orthopedic surgeons still play important roles as gatekeepers while offering reassurances to the families and proper follow-up for mild cases. We emphasize therefore that in cases of confirmed scoliosis in immature patients, those involved in child health care should refer to specialized evaluation without unnecessary delay.

### Patient characteristics

The majority of the patients were girls as reported before [56]. The average angle of trunk rotation (ATR) is in agreement with a previous report [57]. Family history of scoliosis is consistent with earlier studies [11,12,14-16]. Curve classification according to King-Moe compares well with the original publication reporting that type 2 curves were most common and type 5 were the least common [54]. Neurological examination to eliminate underlying neurological pathology was normal in all patients except one patient who was subsequently referred for neurosurgical treatment before brace treatment commenced.

### Scoliosis and back pain

Previous studies have reported slightly increased back pain in adolescents with idiopathic scoliosis compared to the normal population, but the pain is not usually disabling [58,59]. These studies have not showed any association between back pain and curve size, gender, family history of scoliosis, or limb-length discrepancy, but significant association between back pain and mature age and skeletal maturity [58,60]. A recent study showed an association between pain and maturity, overweight and larger proximal thoracic curves [61]. In the present study most patients (59%) reported some, but not disabling back pain and only between 1-3% had back pain almost all the time. One previous study reported less postoperative pain in male patients with AIS [62]. In the present study, we also found a significant association between back pain and girls compared with boys but no association between back pain, curve size, and BMI.

### Comparison of treatment modalities during screening years versus non-screening years

The efficacy of scoliosis screening is under debate [40-44]. To justify screening, it should lead to early detection and initiation of brace treatment at the appropriate time to optimize its efficacy and reduce the option of surgery. Earlier studies suggest that screening may improve the outcome of bracing and either reduce the surgical rate or optimize timing for surgery [43,45,63,64]. A recent case control study reported that screening does not reduce surgery in scoliosis patients [46]. In the present study, we found that the average number of patients braced each year during the period of screening was significantly higher than in the period without screening. Authors clearly acknowledge the methodological weakness, when numbers of those operated were not retrieved from prospectively collected data, but from administrative count data (surgical protocols) over both periods. The Norwegian population has increased during the study years from an average of 4.1 million inhabitants during the screening years to 4.7 million during the non-screening periods, but the population segment of 10–19 year olds who represent the risk population in the study has remained relatively the same (634229 in 1976–1988 to 616715 during 2003–2011) [49]. Within the relatively close periods in comparison, we assume that the prevalence, natural history, and the indications for idiopathic scoliosis treatment have not changed [48]. The p-value in the chi-square statistics comparing the proportions of brace and surgical treatment was statistically significant. Our results therefore suggest that the absence of screening for scoliosis has resulted in less patients being treated with brace and more patients having surgery. However, technical advances in scoliosis surgery in recent years coupled with surgeon attitudes may also contribute to the observed change in treatment trends exhibited over the two periods. The Boston brace has remained the choice of brace type at our institution, but surgical treatment of scoliosis has evolved from the Harrington distraction rods to third generation instrumentation with segmental all pedicle screws construct in the course of the two periods. In addition, during the screening period bracing was administered by one spine surgeon, while different spine surgeons were involved in brace treatment during the period when there was no screening. The issue of non-uniform health care provision has the potential of introducing another bias in the comparison of treatment rates in the two periods.

### Limitations of the study

Resident orthopaedic surgeons with variable experience in scoliosis management participated in the study. The inter-tester reliability was not tested. This variability in experience could influence evaluation of patient characteristics and the recommended treatment. The assessment of back pain applied has not been validated and recording by a surgeon may be less valid than self-assessment by patients. The estimation of surgical rates in the two treatment periods was based on surgical protocols and not on the registration chart at the outpatient clinic. There is an indication that, the number obtained from in the surgical protocol is a more valid estimate of the true number of actually operated than the recorded recommendations. It is also likely that some patients were not registered at the outpatient clinic. In view of the methodological weaknesses and other limitations which the authors clearly acknowledge in the manuscript, the results of the comparison of the rate of brace and surgery treatments during the two periods should be interpreted with caution.

## Conclusion

In the absence of a scoliosis screening program, many patients were referred late and presented with a mean Cobb angle approaching the upper limit of brace treatment indication. The present study suggests that fewer patients are being braced, and more patients are having surgery. However, we acknowledge methodological limitations in comparing treatment from the two periods, and cannot exclude factors other than screening to have contributed to the observed changes. The majority of cases were detected by lay persons and referred too late for specialist evaluation. Scoliosis detected by parents, family and friends were younger than scoliosis detected by the patients themselves. Better dissemination of guidelines for scoliosis referrals are suggested to improve referral timing in the absence of school scoliosis screening programs. The reintroduction of scoliosis screening may be considered in Norway in order to detect idiopathic scoliosis earlier than we do today.

## Competing interests

None of the authors have received benefits for personal or professional use from a commercial party related directly or indirectly to the subject of this manuscript e.g., royalties, stocks, stock options, decision making positions.

## Authors’ contributions

RR, RS and JIB designed the study. RA wrote the drafted manuscript. JIB analyzed the data statistically. All authors were involved in the collection of the data, interpretation of the results, drafting and critical review of the manuscript. All authors have given approval to the final version to be published.
